# Concerns about transmission, changed services and place of birth in the early COVID-19 pandemic: a national survey among Danish pregnant women. The COVIDPregDK study

**DOI:** 10.1186/s12884-021-04108-6

**Published:** 2021-09-30

**Authors:** Katja Schrøder, Lonny Stokholm, Katrine Hass Rubin, Jan Stener Jørgensen, Ellen Aagaard Nohr, Lone Kjeld Petersen, Mette Bliddal

**Affiliations:** 1 Research Unit of User Perspectives, Department of Public Health, University of Southern Denmark, Odense, Denmark; 2 Research Unit of Gynecology and Obstetrics, Department of Clinical Research, Odense University Hospital, University of Southern Denmark, J.B. Winsløws Vej 9, 5000 Odense C, Denmark; 3 OPEN - Open Patient Data Explorative Network, Department of Clinical Research, University of Southern Denmark, and Odense University Hospital, Odense, Denmark

**Keywords:** COVID-19, Place of birth, Pregnancy, Pregnancy-related concerns, Survey study

## Abstract

**Background:**

The outbreak of the COVID-19 pandemic caused great uncertainty about causes, treatment and mortality of the new virus. Constant updates of recommendations and restrictions from national authorities may have caused great concern for pregnant women. Reports suggested an increased number of pregnant women choosing to give birth at home, some even unassisted (‘freebirth’) due to concerns of transmission in hospital or reduction in birthplace options. During April and May 2020, we aimed to investigate i) the level of concern about coronavirus transmission in Danish pregnant women, ii) the level of concern related to changes in maternity services due to the pandemic, and iii) implications for choice of place of birth.

**Methods:**

We conducted a nationwide cross-sectional online survey study, inviting all registered pregnant women in Denmark (*n* = 30,009) in April and May 2020.

**Results:**

The response rate was 60% (*n* = 17,995). Concerns of transmission during pregnancy and birth were considerable; 63% worried about getting severely ill whilst pregnant, and 55% worried that virus would be transmitted to their child. Thirtyeight percent worried about contracting the virus at the hospital. The most predominant concern related to changes in maternity services during the pandemic was restrictions on partners’ attendance at birth (81%). Especially nulliparous women were concerned about whether cancelled antenatal classes or fewer physical midwifery consultations would affect their ability to give birth or care for their child postpartum.. The proportion of women who considered a home birth was equivalent to pre-pandemic home birth rates in Denmark (3%). During the temporary discontinue of public home birth services, 18% of this group considered a home birth assisted by a private midwife (*n* = 125), and 6% considered a home birth with no midwifery assistance at all (*n* = 41).

**Conclusion:**

Danish pregnant womens’ concerns about virus transmission to the unborn child and worries about contracting the virus during hospital appointments were considerable during the early pandemic. Home birth rates may not be affected by the pandemic, but restrictions in home birth services may impose decisions to freebirth for a small proportion of the population.

## Background

During the early months of 2020, the world faced an outbreak of the previously unknown coronavirus disease (COVID-19), which rapidly evolved into a global pandemic. At the pandemic onset, little was known about the virus and its effects on the pregnant woman, the fetus, and the newborn child [1]. As a precaution, pregnant women were labelled together with people at moderate risk for severe illness [2, 3]. They have had to continuously adjust to the evolving evidence, altered recommendations, and restrictions from governments and health authorities. This may have caused great uncertainty and concern for the health and wellbeing of themselves and their children.

The pandemic has created stress and anxiety among pregnant women in different parts of the world [4–6]. Recent studies report that they experience moderate to high levels of psychological distress because they feel unprepared for birth and have fear of perinatal COVID-19 infection [4, 5]. Also, they express concerns about whether restrictions from authorities could disrupt their birth plans [7].

The International Confederation of Midwives (ICM) has raised concerns regarding the human rights of women and their babies by the introduction of alternating protocols for management of pregnancy, birth, and postnatal care in response to the COVID-19 pandemic [8]. In Denmark, women have a statutory right to give birth at home and receive midwifery care, free of charge [9]. However, an amendment to the Danish Epidemic Act, authorised by the Minister for Health, led to discontinued home birth services in four out of five Danish regions by March 2020. Similar actions have been taken in other countries, mainly as a result of staff shortages and service pressures in maternity and related services during the COVID-19 pandemic. Due to this reconfiguration of health care services during the COVID-19 pandemic, there are anecdotal reports that an increased number of women would choose to have an unassisted birth in the COVID-19 pandemic due to the reduction in birthplace options [7, 10].

We conducted a national survey among Danish pregnant women (COVIDPregDK) in April and May 2020. The objective of this study was to investigate i) the level of concern about coronavirus transmission in Danish pregnant women, ii) the level of concerns related to changes in maternity services due to the pandemic, and iii) implications for their choice of place of birth during the pandemic.

## Methods

We conducted a nationwide cross-sectional study using survey data on all registered pregnant women (gestational age ≥ 12 weeks) on April 24, 2020. We used the data to describe concerns regarding pregnancy and birth in relation to the COVID-19 pandemic.

### Setting

Denmark was one of the first countries to initiate a National lockdown with closed institutions and public workplaces on March 11, 2020 [11], the same day as the World Health Organization declared the pandemic [12]. All non-critical hospital contacts were postponed or cancelled. As of mid-April, Denmark started a controlled reopening with cautiously increasing in access to the health care system besides COVID-19 related contacts [13]. In the early months of the pandemic and during the study period, test capacity was limited. Very few pregnant women tested positive for COVID-19, i.e. by April 22, 2020, 1245 pregnant women had been tested and 66 tested positive [14].

Maternity services in Denmark were considered as critical functions. Antenatal or outpatient appointments were continued or replaced with video consultations. Antenatal classes were cancelled or replaced with online classes or videos. The woman’s partner was not allowed to participate in ante- or postnatal appointments, including ultrasound scans, and he or she was only allowed in the labour ward if they had no symptoms of COVID-19. Home birth services were temporarily discontinued in four out of five regions.

### Study population and material

The Danish National Patient Registry is a nationwide hospital register which is used extensively for research. It contains information on all hospital contacts in Denmark and, due to the Danish tax-funded health care system, virtually all pregnancies and childbirths are registered [15, 16]. For this study, we invited women if they were registered with a nuchal translucency scan conducted in gestational week 12 and other ultrasound scan performed during pregnancy. To include only women pregnant at the time of the survey, we excluded women with a diagnosed miscarriage, induced abortion, or childbirth after the inclusion date. The study population was defined at April 24, 2020, and the invitation to participate was sent the same day. Each woman received a link to a web-based questionnaire using the Danish secure digital solution (e-boks) that allows contacts based on a unique personal identification number, assigned to all inhabitants at birth or first immigration [17]. A total of 30,009 pregnant women were invited to participate. Women who had not filled the questionnaire within 14 days, received a reminder with a new link to the survey. The data collection ended on May 24, 2020. We disregarded partly completed questionnaires if only basic characteristic questions were answered (question 1–21, [18]). All other replies from partly completed questionnaires were used.

We constructed a questionnaire in March 2020 aimed at pregnant women during the early COVID-19 pandemic. It included questions on COVID-19 worries related to pregnancy and birth, worries related to experienced effects of changes in maternity services, and finally questions on considerations regarding the place of birth. These three dimensions are the focus of this study, and the items were developed specifically for this study. Due to the exceptional circumstances of the lockdown, the time frame for the development of these dimensions did not allow for piloting or testing for internal consistency. The questionnaire also covered demographics, general health, COVID-19 symptoms and testing, and mental health, including anxiety and stress. It can be seen in full in the baseline article of the COVIDPregDK study [18].

### Covariates

From the COVIDPregDK survey, we retrieved background information on age and parity (0/≥1) as well as information on any positive COVID-19 tests in the woman’s household prior to the survey.

Concerns about the transmission of COVID-19 during pregnancy and birth were addressed in items related to various potential transmission situations framed as: “To what extent are you worried about…..?” with the following five response categories: “To a great extent, to some extent, to a small extent, not at all, or don’t know”. Similarly, the items on concerns on changes in maternity services included questions on worries of not having antenatal healthcare visits, changes in settings, fewer possibilities in childbirth, including restrictions on the presence of a partner. These items held the same response categories as above.

Finally, considerations on place of birth were addressed in a series of items on home birth. To give the women the possibility to reply as accurately as possible, the response categories for these items varied accordingly.

### User involvement

During and after the development of the questionnaire, a panel of six pregnant women (gestational age, range 6–38 pregnancy weeks) filled out the questionnaire and gave their feedback, resulting in a clarification of questions and a more user-friendly layout.

### Statistical analysis

To characterize the study population, we used statistics presenting means with standard deviations on continuous data and distribution of frequencies for categorical data on demographics and baseline data. To illustrate the distribution of answers to each question, we tabulated each reply and presented them as plotted figures with information on numbers and percentages. We performed the descriptive analyses according to parity since answers were expected to differ between nulliparous (nullips) and multiparous women (multips) and tested for difference by chi2 tests. *P*-values < 0.05 were regarded as statistically significant.

## Results

A total of 17,995 women participated in the survey (16,147 completed the full questionnaire, 1848 completed partly). The response rate was 60%. The participating women had a mean age of 30 years, with a slight majority being multiparous (52%). For characeteristics (smokers, BMI, education, employment and chronic diseases) on study population, see Table 1. Only very few women reported to have been tested positive for COVID-19 or to be living with a person tested positive by the time they filled the questionnaire (*n* = 52 and 35, respectively). To be noted, during most of the data collection period, the availability of tests in Denmark was limited and restricted to severe cases with symptoms of COVID-19 that required treatment.

**Table 1:** Characteristics of study population

	Baseline characteristics
Total (n)	17995
Age (median (IQR))	30.0 (28.0;34.0)
Parity	
Nulliparous	8581 (47.7)
Current smoker	548 (3.1)
Pre-pregnancy body mass index, median (IQR)	23.5 (21.2;27.0)
Education	
Short cycle higher education	1353 (7.5)
Medium cycle education	6194 (34.4)
Long education	5532 (30.7)
Other	4916 (27.3)
Job situation	
Employement	16262 (90.4)
Unemployement	911 (5.1)
Long-term absence of leave, not pregnancy-related	208 (1.2)
Other status	614 (3.4)
Chronic diseases	2909 (16.5)
History of mental illness	2252 (12.8)
Footnotes	
*N missing*	*Age n<5, Parity 19, Current smoker 403, BMI 507, Education 0, Job situation 0, Chronic disease 335, Psychiatric disease 338*

### Concerns about transmission of COVID-19 during pregnancy, birth and postpartum

**Fig. 1:**
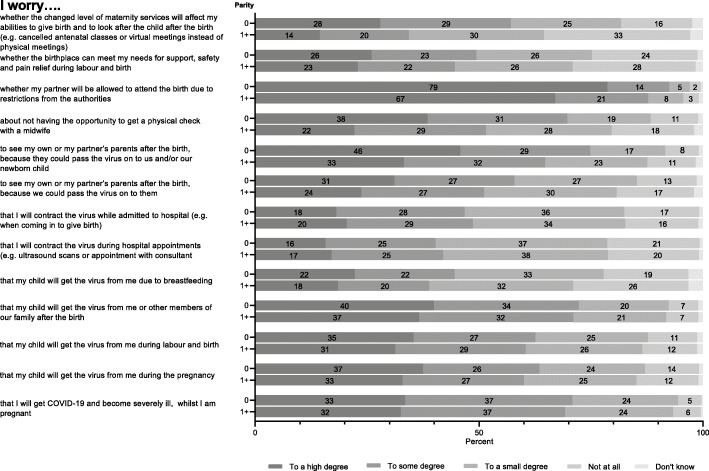
Level of concern about transmission of COVID-19 during pregnancy and birth and level of concern regarding changes in maternity services (nullipara (0): *n* = 7832, multipara (1+): *n* = 8254)

Figure 1 shows the participants’ level of concern about transmission of COVID-19 during pregnancy and birth. Sixty-three percent (nullips; 65%, multips; 61%) worried about getting COVID-19 and becoming severely ill, whilst they were pregnant, and 55% (nullips; 58%, multips; 53%) worried that their child would get the virus from them during the pregnancy or during labour and birth. Two-thirds worried that their child would get the virus from themselves or other family members after the birth, and concerns about contact with grandparents were predominant. Fewer (37%) worried that their child would get the virus from them due to breastfeeding. Just over one third (38%) worried that they would contract the virus during hospital appointments or while admitted to hospital (e.g. when giving birth). Generally, nulliparous women seemed to have a modestly higher level of concern about transmission of COVID-19 to their newborn child, whereas multiparous women tended to worry more about contracting the virus during pregnancy than nulliparous women (*p*-values < 0.05 except for worries for infection due to hospital contacts, p-values 0.06).

### Concerns about changed maternity services

Regarding concerns related to changes in maternity services, we observed a considerable difference between nulliparous and multiparous women (Fig. 1). Fifty-four percent (nullips; 64%, multips; 46%) worried about not having the opportunity to get a physical check with a midwife, and 41% (nullips; 52%, multips; 30%) worried whether the changed level of maternity services would affect their prerequisite or abilities to give birth and care for the child after the birth (e.g. cancelled antenatal classes or virtual meetings instead of physical meetings). A substantial concern for both nulliparous and multiparous women was the risk of giving birth without their partner being present due to restrictions from the authorities; 81% (nullips; 84%, multips; 78%) expressed such worries. All *p*-values were statistically significant.

### Considerations about place of birth during the pandemic

All participants were asked whether they had planned a home birth prior to the pandemic, and 81.5% replied *no* (*n* = 14,677), 3.5% replied *yes* (*n* = 659), 1% replied *yes, but conditions related to my pregnancy has led to professional advice against home birth* (*n* = 173), 3.3% had still not decided (*n* = 586), and 0.5% did not know (*n* = 92). In the group of women who opted for a home birth, only 26% were nulliparous, and significantly more had a medium or long education than the women opting for a hospital birth. For characeteristics (smokers, BMI, education, employment and chronic diseases) on study population according to place of birth preference, see Table 2. Due to the temporary discontinuation of home birth services in most regions, participants were asked whether they presently had access to public midwifery services at a home birth. Twenty-five percent did not have access due to the lockdown, 19% did, and 46% did not know.

**Table 2:** Characteristics of study population according to place of birth preference

Did you plan to give birth at home prior to the pandemic?	Yes (homebirth)	No (hospital)	I still have not decided	Yes, but conditions related to my pregnancy has led to professional advice against home birth	*P*-value
Total (n)	659	14677	586	173	
Age (median (IQR))	31.0 (29.0;34.0)	30.0(28.0;34.0)	30.5 (28.0;34.0)	32.0(29.0;34.0)	<0.001*
Parity					
Nulliparous	170(25.8)	7312(49.9)	279(47.6)	37(21.5)	<0.001*
Current smoker	8(1.2)	437(3.0)	14(2.4)	<5	0.243**
Pre-pregnancy body mass index, median (IQR)	22.8(20.7;25.9)	23.5(21.2;27.0)	23.2(21.1;26.1)	23.7(21.5;27.2)	<0.001*
Education					0.005**
Short cycle higher education	35(5.3)	1103(7.5)	38(6.5)	11(6.4)	
Medium cycle education	261(39.6)	5118(34.9)	208(35.5)	67(38.7)	
Long education	234(35.5)	4676(31.9)	192(32.8)	59(34.1)	
Other	129(19.6)	3780(25.8)	148(25.3)	36(20.8)	
Job situation					<0.001**
Employement	580(88.0)	13381(91.2)	513(87.5)	142(82.1)	
Unemployement	35(5.3)	726(4.9)	38(6.5)	14(8.1)	
Long-term absence of leave, not pregnancy-related	7(1.1)	174(1.2)	<5	<5	
Other status	37(5.6)	396(2.7)	32(5.5)	13(7.5)	
Chronic diseases	77(11.7)	2502(17.0)	76(13.0)	49(28.3)	<0.001**
History of mental illness	97(14.7)	1896(12.9)	88(15.0)	32(18.6)	0.041**
Footnotes					
*N missing: homebirth*	*Age 0, Parity 0, Current smoker 0, BMI n<5, Education 0, Job situation 0, Chronic disease 0, Mental illeness 0*				
*N missing: hospital*	*Age 0, Parity 11, Current smoker 0, BMI 68, Education 0, Job situation 0, Chronic disease 0, Mental illeness 0*				
*N missing: undecided*	*Age 0, Parity 0, Current smoker 0, BMI 0, Education 0, Job situation 0, Chronic disease 0, Mental illeness 0*				
*N missing: yes, but there...*	*Age 0, Parity n<5, Current smoker 0, BMI 1, Education 0, Job situation 0, Chronic disease 0, Mental illness 0*				

*Nonparametric equality-of-medians test

**chi2 test

**Fig. 2:**
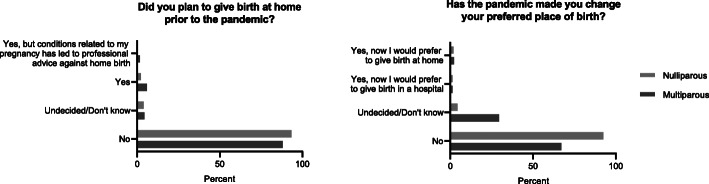
Planned place of birth prior to the pandemic and change of preference due to the pandemic (nullipara (0): n = 7832, multipara (1+): *n* = 8345)

Participants were asked whether the pandemic had changed their preferred place of birth, which 3,5% (*n* = 651) agreed to. In this group, 254 women would now prefer giving birth at home instead of the hospital, and 397 would now prefer giving birth in a hospital instead of at home (Fig. 2).

**Fig. 3:**
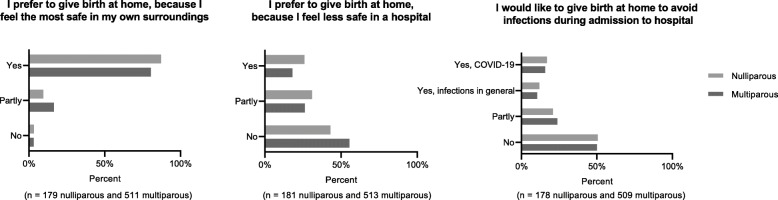
Reasons for choosing a home birth

Three percent stated that they planned to give birth at home during the present circumstances (*n* = 534), 1% were undecided (*n* = 164). Of the 698 women who considered a home birth, 81% preferred to give birth at home because they felt most safe in their own surroundings, whilst 20% preferred a home birth because they felt less safe in a hospital. Half of the women considering home birth (*n* = 349) stated that avoiding infections in the hospital was a part of the reason for their decision, and only 16% specifically stated COVID-19 infection as a reason (Fig. 3).

**Fig. 4:**
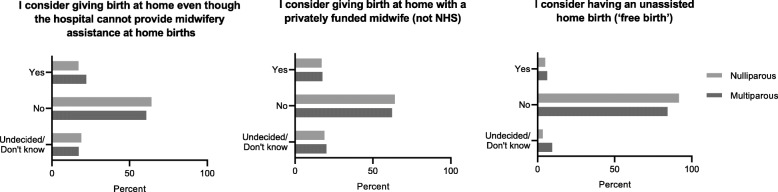
Choice of midwifery assistance for home birth (*n* = 698)

Of the 698 women who considered a home birth, 31 (17%) nulliparous and 114 (22%) multiparous women considered giving birth at home despite the discontinued midwifery services due to COVID-19 restrictions, and 123 (18%) were undecided (*p*-value 0.16) (Fig. 4). A total of 125 (18%) women considered a home birth with a private midwife, and 135 (19%) were undecided (p-value 0.67). Nine (5%) nulliparous and 32 (6%) multiparous women considered having an unassisted home birth (‘free birth’), and 55 (7%) women were undecided (p-value 0.02).

## Discussion

In this national survey among Danish pregnant women on concerns about pregnancy and birth in the early COVID-19 pandemic, 17,995 women (60%) participated. Concerns of transmission were considerable and related to getting severely ill and transmitting the virus to the unborn child. Just over one third worried that they would contract the virus during hospital appointments or while admitted to hospital (e.g. when giving birth). Concerns related to changes in maternity services during the pandemic were significant and even more among nulliparous than multiparous women. Nulliparous women were especially worried about whether the changed level of maternity services would affect their abilities to give birth and care for their child after birth. A predominant concern for all women was whether their partner would be allowed to attend the birth. In the subcohort of women planning to give birth at home, one in five considered a home birth assisted by a private midwife and a small proportion considered giving birth with no midwifery assistance at all. Feeling safe in own surroundings was a more prevalent reason for planning a home birth than fear of nosocomial infections.

This is the first population-based study to investigate pregnant women’s concerns about pregnancy and birth during the first wave of the COVID-19 pandemic and lockdown measures in Denmark. This study has a registerbased inclusion of pregnant women and the Danish secure digital solution (e-boks) allowed contact and invitation to virtually all relevant potential participants. We consider this to be a strength compared to studies recruiting women via social media, but with a response rate of 60%, selection bias must also be considered in this study. We acknowledge that this study is purely descriptive and statistics are used accordingly. Our aim was to describe our study population on concerns related to the pandemic and pregnancy and implications for women’s choice of birth place. Due to the very large sample size, test for differences between nulli- and multiparous women, were mostly statistically significant, however the clinical relevance was regarded of little importance unless stated.

At the time of our data collection, much was still unknown about the virus and its effects on both the pregnant woman, the fetus, and the newborn child [1], leaving pregnant women in a state of great uncertainty and concern. Since strong patient-provider relationships have been shown to reduce distress in pregnant women and result in better self-care [19], maternity services should be continued even during a pandemic. Effective use of telehealth appointments may support continuity of care and the patient-provider relationship. Healthcare professionals should pay attention to pregnant women’s concerns about transmission to the unborn child and their worries about contracting the virus during hospital appointments or while admitted to the hospital that was evident in this study. A study examining the association between number of healthcare visits and SARS-CoV-2 infection in obstetrical patients suggests that healthcare visits are not likely to be an important risk factor for infection [20]. Antenatal and postnatal care should be regarded as essential, and women encouraged to attend [2], whilst observing current physical distancing measures as recommended by local and national authorities.

Although a generally high level of concern about the consequences of the changed level of maternity services was found in this study, it was more pronounced in the group of nulliparous women. Childbearing, from conception until after the birth of a woman’s first child, is a major life event that can bring about many challenges for the woman and her family [21, 22]. Contact with other pregnant women and new mothers has been shown to be a vital source of support for women, and one way of making contact with other expectant mothers is through antenatal classes [21–23]. Feeling well-supported in pregnancy has been shown to reduce the likelihood of low-birthweight infants, increase self-confidence in the motherhood role, and reduce the prevalence of depressive symptoms in the postpartum period [21, 24]. Conversely, women who do not have the opportunity to draw on ‘mother-to-mother talk’ as a source of support may find themselves feeling isolated and with low mood [21]. Consequently, redesigning maternity services during the global pandemic is a delicate balance between reducing the risk of transmission and maintaining a sufficient level of continuity of care and support. The importance of facilitating both antenatal classes and postnatal groups should be recognized as a cancellation, or online meetings may hinder new mothers (and fathers or co-parents) in building beneficial, supportive networks with peers.

The support of the partner during labour and childbirth seems to be of paramount importance as an encourager and to give the woman confidence in her ability to get through the labour [21]. In this study, the greatest concern of all was the risk of giving birth without the partner being present due to restrictions from the authorities. This emphasises that childbirth is regarded as a shared life event between the woman and her partner and that women rely heavily on the support of their partner, who have been described as their *co-pilot,* a voice of reason and an advocate who will speak up for their rights and wishes in case they are not able to make decisions themselves [23].

Another restriction from the authorities during the first lockdown in March, April and May 2020 was the temporary discontinuation of home birth services in four out of five regions in Denmark. This measure affected a smaller proportion of the participants since 4% (*n* = 659) had planned to give birth at home before the pandemic, and 3% (*n* = 586) were undecided. However, the impact of this restriction was so distressing that a small group either considered or were undecided about giving birth at home regardless either assisted by a privately funded midwife or without midwifery assistance. This stresses that maternity services need to provide support for all pregnant women, regardless of their reasons for birthplace preferences during a pandemic to prevent them from withdrawing from maternity services.

Rapid-response articles have suggested that the COVID-19 pandemic will lead to an increase in births at home or in smaller units outside the hospital [7, 10, 25]. Davis-Floyd et al. [25] argue that US hospitals were are now being perceived as sites of contagion more than ever before, and they pose the questions: Are pregnant women becoming frightened of hospital-based care, and is that fear starting to outweigh their fear of out-of-hospital birth? Preis et al. have found that women who preferred a community birth had less fear of childbirth, stronger beliefs that birth is a natural process and were more concerned about contracting COVID-19 in the hospital [26]. In this study, 1.4% stated to have changed their mind from home birth to hospital birth, and 2.2% from hospital to home birth. Hence, the total movement from hospital to home birth is 0.6% at this intentional stage. However, the decision-making may still be altered according to changes in individual, obstetrical, or pandemic circumstances. These findings should be interpreted with caution as numbers were few. In contrast to US findings [26], our results indicate that the pandemic has not affected Danish women’s choices greatly at this point. The proportion of pregnant women who considered a home birth is equivalent with the present home birth rate in Denmark, which has risen from 0.9% in 2011 to 3.3% in 2018 [27]. This may indicate a high level of general trust in the Danish healthcare system and the authorities during the first confusing months of the COVID-19 pandemic.

From a global perspective, the pandemic has different consequences in different healthcare settings, and these Danish findings may not be transferable to low and middle-income countries. However, pregnant women, infants and new mothers are decidedly vulnerable in times of disasters and crisis in all parts of the world. According to WHO, all pregnant women and their newborns, including those with confirmed or suspected COVID-19 infections, have the right to high quality care before, during and after childbirth, including mental health care. This includes being treated with respect and dignity, having a companion of choice present during delivery, clear communication by maternity staff, appropriate pain relief strategies, mobility in labour and birth position of choice [28]. ICM recommends that protocols for pregnancy and childbirth during the COVID-19 pandemic are evidence-based, that every woman has the right to information, to give consent, to refuse consent and to have her choices and decisions respected and upheld. This includes the right to have a companion of her choice with her during her labour and birth. They also state that in countries where the health systems can support homebirth, healthy women experiencing a normal pregnancy and with support from qualified midwives, with appropriate emergency equipment, may be safer birthing at home or in a primary maternity unit/birth centre than in a hospital where there may be many patients (even non-maternity patients) with Covid-19 [8]. There is a need for further research in how the pandemic, the derived change in maternity services, and the level of concern for the health and wellbeing of themselves and their children may impact the mental health of pregnant women.

If COVID-19 is suspected or confirmed, health workers should take all appropriate precautions to reduce risks of infection to themselves and others, including hand hygiene, and appropriate use of protective clothing like gloves, gown and medical mask.

## Conclusion

Concerns of transmission and concerns related to changes in maternity services during the pandemic, including restrictions on partners’ attendance at birth, were considerable, especially for nulliparous women in the early COVID-19 pandemic. The proportion of women who considered a home birth during the first lockdown in March and April 2020 was equivalent to the present home birth rate in Denmark. However, despite temporary discontinue of home birth services, a small proportion considered a home birth either assisted by a privately funded midwife or with no midwifery assistance at all.

## Data Availability

The data that support the findings of this study are available from the corresponding author upon reasonable request and after obtaining adequate permission according to Danish law.

## Abbreviations

Nulliparous women: nullips
multiparous women: multips
